# Endoplasmic Reticulum Stress and Unfolded Protein Response in Cartilage Pathophysiology; Contributing Factors to Apoptosis and Osteoarthritis

**DOI:** 10.3390/ijms18030665

**Published:** 2017-03-20

**Authors:** Alexandria Hughes, Alexandra E. Oxford, Ken Tawara, Cheryl L. Jorcyk, Julia Thom Oxford

**Affiliations:** 1 Department of Biological Sciences, Boise State University, Boise, ID 83725, USA; alexhughes1@u.boisestate.edu (A.H.); aliceoxford@u.boisestate.edu (A.E.O.); cjorcyk@boisestate.edu (C.L.J.); 2 Biomolecular Sciences Graduate Program, Boise State University, Boise, ID 83725, USA; kentawara@u.boisestate.edu; 3 Biomolecular Research Center, Boise State University, Boise, ID 83725, USA

**Keywords:** chondrocyte, osteoarthritis, endoplasmic reticulum (ER) stress, unfolded protein response, endochondral ossification

## Abstract

Chondrocytes of the growth plate undergo apoptosis during the process of endochondral ossification, as well as during the progression of osteoarthritis. Although the regulation of this process is not completely understood, alterations in the precisely orchestrated programmed cell death during development can have catastrophic results, as exemplified by several chondrodystrophies which are frequently accompanied by early onset osteoarthritis. Understanding the mechanisms that underlie chondrocyte apoptosis during endochondral ossification in the growth plate has the potential to impact the development of therapeutic applications for chondrodystrophies and associated early onset osteoarthritis. In recent years, several chondrodysplasias and collagenopathies have been recognized as protein-folding diseases that lead to endoplasmic reticulum stress, endoplasmic reticulum associated degradation, and the unfolded protein response. Under conditions of prolonged endoplasmic reticulum stress in which the protein folding load outweighs the folding capacity of the endoplasmic reticulum, cellular dysfunction and death often occur. However, unfolded protein response (UPR) signaling is also required for the normal maturation of chondrocytes and osteoblasts. Understanding how UPR signaling may contribute to cartilage pathophysiology is an essential step toward therapeutic modulation of skeletal disorders that lead to osteoarthritis.

## 1. Introduction

Long bones in the vertebrate body develop through the process of endochondral ossification. This process begins with the condensation of mesenchymal stem cells and the differentiation of these cells into chondrocytes. Maturation of the growth plate ensues with the rapid proliferation and columnar organization of committed chondrocytes (for review, see Kozhemyakina 2015) [1]. Chondrocytes near the center of the cartilage anlagen enter a pre-hypertrophic stage and terminally differentiate into hypertrophic chondrocytes, which commence the process of mineralization through the secretion of alkaline phosphatase (ALP) and other essential matrix proteins. Osteoprogenitor cells invade the cartilage intermediate and differentiate into osteoblasts, further transitioning the template into bone. The physical properties of cartilage and bone can be attributed to the array of structural extracellular matrix (ECM) proteins secreted by both chondrocytes and osteoblasts.

Of particular relevance to endochondral bone formation are the collagens. The collagens are structural, extracellular matrix (ECM) proteins that fulfill differing roles throughout the course of chondrogenesis. All of the 28 known collagens are trimeric triple-helices. Some collagens are homotrimers, such as type II collagen (α1(II)_3_), which is solely encoded by the gene *COL2A1*. Several other collagens are heterotrimers and are thus derived from the products of two or three distinct genes. Many intermediary steps lay between the initial translation of procollagen monomers and the secretion of complete collagen trimers into the ECM. Consequently, there are ample opportunities for collagen biosynthesis and assembly to go awry. Perhaps most significantly, deleterious polymorphisms in collagen-encoding genes may impact the proper folding, assembly, or function of collagens. The integrity of a connective tissue certainly depends upon the proper synthesis of its structural constituents. However, recent perspectives have revealed that collagen misfolding has consequences for not only the extracellular microenvironment, but also for the intracellular homeostasis of chondrocytes and osteoblasts. In particular, the aggregation of unfolded or misfolded proteins in the endoplasmic reticulum (ER) lumen may induce a conserved signaling pathway, the unfolded protein response (UPR). The UPR functions to restore homeostasis by (1) upregulating chaperone proteins to assist with folding; (2) preventing the translation of non-UPR proteins; and (3) initiating apoptosis when resolution is unlikely. The UPR has a pathogenic role in several neurodegenerative diseases, type II diabetes, and non-alcoholic fatty liver disease [2], where signaling may contribute to the apoptosis of essential, mature cell types. However, unlike many of the cell types affected in such diseases, chondrocytes and osteoblasts undergo physiological UPR signaling in the course of normal maturation. Thus, UPR signaling has dually essential and detrimental roles in the bone development of individuals with congenital collagenopathies and chondrodysplasias.

## 2. Aetiology of Chondrodystrophies with Higher Risk of Osteoarthritis

Collagens type I, II, III, V, XI, XXIV, and XXVII are described as the fibrillar collagens due to their formation of collagen fibrils in the ECM [3,4,5,6]. Of these, the fibrillar collagens are further classified by their relative abundance in fibrils—types I, II, and III are referred to as the major fibrillar collagens, while types V and XI are quantitatively minor [6,7]. Sequence analysis predicts that types XXIV and XXVII form a third clade of fibrillar collagen genes [3,4,8]. In addition to sharing the functional role of fibril-formation, the fibrillar collagens share several structural characteristics. Collagens type V, XI, XXIV, and XXVII share a thrombospondin-N-terminal-like (TSPN) motif, adjacent to the variable region (VR) [3,9]. The α1 polypeptide of collagen XXIV, α1(XXIV), shares structural similarity with α1(V), α1(XI), and α2(XI) [3]. Furthermore, deleterious mutations in these genes may result in phenotypically similar outcomes. A mutation in the recently-discovered *COL27A1* has been reported in a Puerto Rican population with Steel syndrome (ORPHA:438117; OMIM #615155), a chondrodysplasia that shares multiple symptoms with Stickler syndrome (ORPHA #828) [10].

## 3. Individuals with Chondrodystrophies Frequently Develop Early-Onset Osteoarthritis

While mutations in fibrillar collagens such as types II and XI are associated with chondrodystrophies, mutations in fibril-associated collagen with interrupted triple helices (FACIT) that associate with fibrillar collagens such as collagen IX, have also been established as contributing and causative factors. The assembly of collagen II, XI, and IX into a cross-linked heteropolymer forms the core fibrillar network of articular cartilage, accounting for two-thirds of the dry mass of adult articular cartilage [11,12,13]. Disruptions to this network may render articular cartilage more susceptible to mechanical damage and contribute to the progression of osteoarthritis (OA) [13]. Indeed, mutations in *COL2A1*, *COL11A1*, *COL11A2*, *COL9A1*, *COL9A2*, and *COL9A3* have been associated with Stickler syndrome and early-onset OA [14,15]. Approximately 50% of individuals with Stickler syndrome will develop OA prior to the age of 30 [16]; however, the signaling events involved in its onset remain speculative.

In age-related OA, the formation of advanced glycation end-products (AGEs) and the binding of AGEs to the receptor for AGEs (RAGE) expressed by articular chondrocytes are considered to be contributing factors that underlie the progression of OA [17]. AGEs contribute to the degradation of type II collagen via the upregulation of matrix metalloproteinase-13 (MMP-13) [18], the induction of ER stress signaling [19,20,21], and initiation of inflammatory NF-κB and MAPK signaling [19,22]. Although AGE/RAGE activity in articular cartilage has been shown to increase with age, Larkin et al. (2013) demonstrated that, following knee destabilization surgery, young RAGE-knockout mice were protected from developing OA [23]. Compared to WT mice, the RAGE-knockout mice displayed an attenuated response to the surgery, as quantified by the OA biomarkers MMP-13, high temperature requirement protease A-1 (HtrA-1), and discoidin domain receptor-2 (Ddr-2) [23]. These findings suggest that AGE/RAGE signaling may contribute to OA in both early and later stages of life, rendering AGE/RAGE signaling a potential contributor to precocious OA.

As MMP-13 degrades type II collagen and may thus disrupt the integrity of articular cartilage, its central importance in OA progression has been well-defined. The use of several mouse models of OA has demonstrated that many pathways of OA progression converge on MMP-13 expression and activity (for review, see Goldring et al. 2011) [24]. Analyses of articular cartilage in the cho (chondrodysplasia) mouse have provided insight into how disruptions of a core fibrillar network collagen chain, COL11A1, may precipitate progressive cartilage destruction and the progression of osteoarthritis. The neonatal-lethal, homozygous cho/cho mouse features a severe chondrodysplasia caused by a point deletion in the *COL11A1* gene, leading to a reading frame shift and the premature termination of the translation of the COL11A1 protein [25]. However, the heterozygous cho/+ mouse survives without apparent skeletal defects at birth, but develops OA of the knee and temporomandibular joints at three months [26]. At six months, Xu et al. (2005) reported a six-fold increase in MMP-13 activity in cho/+ mice, when compared to WT littermates. This increase in MMP-13 activity was concomitant with the upregulation of Ddr2. As the cho/+ mouse displays thicker collagen II fibrils than the WT mouse, it has been postulated that the increased proximity of articular chondrocytes to collagen II permits collagen II to interact with cell-surface Ddr2, for which collagen II is its natural ligand [26,27]. This key event allows for the downstream activation of MMP-13 transcription and the subsequent proteolysis of type II collagen.

Another mouse model of conditions resulting in osteoarthritis [28,29] is the disproportionate micromelia (Dmm) mouse. The Dmm mouse is characterized by a three-nucleotide mutation in the C-propeptide domain of COL2A1 [30,31]. Unlike the cho/+ mouse, the heterozygote Dmm/+ mouse does exhibit mild dwarfism at birth; however, both develop OA, beginning at three months of age. Additionally, the progression of OA in the knees of both cho/+ and Dmm/+ mice involves the Ddr2-HtrA-1-MMP-13 signaling axis [26,28]. Uniquely, it has been demonstrated that the knee articular chondrocytes of Dmm/+ mice display a distended rough endoplasmic reticulum (rER) and evidence of ER stress-induced UPR signaling at birth, prior to the development of OA [28]. This was thought to have resulted from the intracellular retention of misfolded mutant COL2A1. It has additionally been demonstrated that UPR-induced genes, such as the C/EBP homologous protein (CHOP) [32], may contribute to the apoptosis of articular chondrocytes in the progression of OA.

## 4. A Role for the Unfolded Protein Response in Chondrodystrophies and Early Onset OA

**Figure 1:**
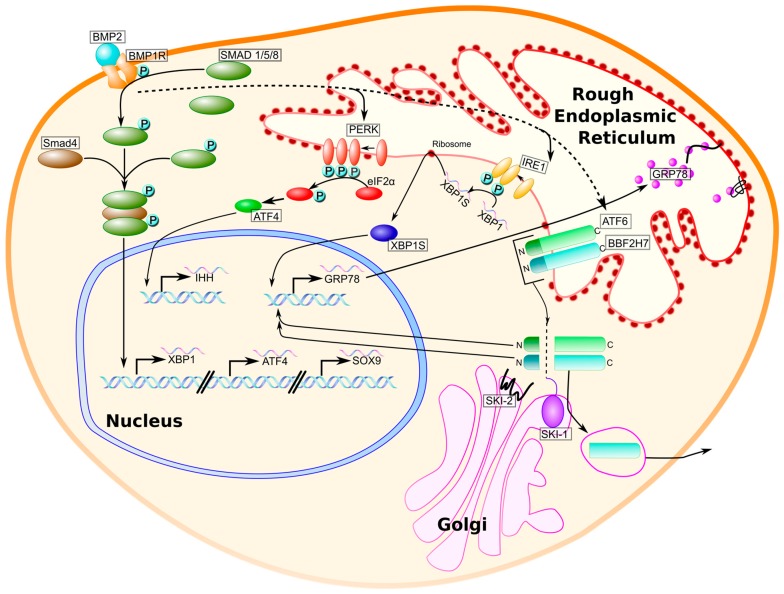
BMP-mediated induction of physiological unfolded protein response (UPR) signaling in chondrocytes. Bone morphogenetic protein 2 (BMP2) is an extracellular growth factor that enacts UPR activity in chondrocytes in two ways: (1) the binding of BMP2 to its receptor, BMP1R, results in Smad 1/5/8 phosphorylation and the association with a co-Smad, Smad4, to form a trimeric activator of UPR genes *XBP1*, *ATF4*, and the chondrogenesis regulator *SOX9* (solid line); and (2) BMP2 increases the activity of the UPR transducers PKR-like endoplasmic reticulum kinase (PERK), inositol-requiring endonuclease 1 (IRE1), activating transcription factor 6 (ATF6) (dotted line), and BBF2H7, an ATF6 homolog expressed by chondrocytes. PERK phosphorylates eukaryotic translation initiation factor 2a (eiF2a) to halt general protein translation while promoting the translation of activating transcription factor 4 (ATF4) (translation not shown). IRE1 splices XBP1 mRNA to generate XBP1S mRNA, which is translated to yield the transcription factor XBP1S. ATF6 and BBF2H7 translocate to the Golgi, where they are cleaved by site-1 and site-2 proteases (SKI-1 and SKI-2) to generate N-termini that serve as transcription factors for chaperone proteins, such as glucose-regulated protein 78 kDa (GRP78). The upregulation of chaperone proteins enhances the folding capacity of the ER during chondrogenesis.

The unfolded protein response (UPR) is a three-armed signaling pathway induced by the burden of accumulated unfolded or misfolded proteins in the endoplasmic reticulum (ER), a condition referred to as ER stress [33,34]. Figure 1 illustrates the orchestration of this pathway, which is intended to restore homeostasis to the ER by attenuating protein translation, upregulating ER-resident chaperone proteins, and initiating apoptosis when ER stress is sustained [35,36]. In homeostatic conditions, the chaperone protein glucose-regulated protein, 78 kDa (GRP78, also known as BiP/HSPA5), is bound to the ER transmembrane stress sensors inositol-requiring endonuclease 1 (IRE1), activating transcription factor 6 (ATF6), and PKR-like endoplasmic reticulum kinase (PERK) [36]. When unfolded or misfolded proteins are present in the ER lumen, bound GRP78 dissociates from the three ER stress transducers, to bind exposed hydrophobic residues. The disruption of GRP78-transducer interactions is initiative of downstream signaling through each of the three arms of the UPR [36,37].

UPR signaling is involved in the progression of OA. During development, however, it also negatively impacts chondrocyte proliferation and the longitudinal growth of long bones, independently of ECM abnormalities [38]. The Dmm/+ mouse displays UPR activation in articular chondrocytes prior to the onset of OA [28]. Additionally, many genetic chondrodysplasias that affect both collagenous and noncollagenous ECM proteins involve the UPR mediation of resultant misfolded proteins. The best-studied mutations that induce UPR activity are those of *COL2A1*, *COL10A1*, the cartilage oligomeric matrix protein (*COMP*), and matrilin-3 (*MAT3*) (for review, [39]). Additionally, several pathogenic variants in the fibrillar collagens result in UPR activation in chondrocytes (Table 1). Mutations in the genes encoding α chains for many of the fibrillar collagens have been reported to induce ER dilation or the UPR in the cell types responsible for their secretion. ER dilation may be readily observed by electron microscopy and is accepted as an indicator of ER stress in myriad cell types, including pancreatic β-cells [40], hepatocytes [41], and several tumor cell lines [41,42,43]. While it has been demonstrated that mutations in *COL2A1* lead to UPR activation, this has not been established for *COL11A1*, *COL11A2*, *COL9A1*, *COL9A2*, or *COL9A3*. However, the fibrillar collagen genes *COL11A1* and *COL11A2* share several structural characteristics with other fibrillar collagens, many of which are included in Table 1. Whether mutant fibrillar collagens retain the capacity to fold correctly requires an understanding of how collagen biosynthesis proceeds under normal conditions.

## 5. Collagen Biosynthesis and the Unfolded Protein Response in Chondrodysplasia

Collagen biosynthesis, folding, and assembly have been best-studied processes in type I collagen, but these contributions likely extend to other collagens as well. As the ribosomal translation of a collagen chain proceeds, the nascent procollagen polypeptide extends into the lumen of the rough endoplasmic reticulum (rER), coincident with synthesis [49,50]. Procollagen chains consist of a collagenous domain flanked by N- and C-propeptides. The first domain to enter the rER lumen, termed the N-propeptide, is followed by the continuing synthesis of the collagenous domain. The collagenous domain, conserved among all collagens, consists of Gly-X-Y repeats and is subject to extensive posttranslational modification. Prolyl 4-hydroxylases (P4Hs), prolyl 3-hydroxylases (P3Hs), and lysyl hydroxylases (LHs) hydroxylate proline residues (P4H and P3H) and lysine residues (LHs), the latter of which is crucial for the eventual glycosylation and cross-linking of collagen molecules in fibril formation [49]. For many proteins, folding begins at the N-terminus as translation continues, but this is not the case for procollagens [51]. Instead, folding initiates following the association of participating C-terminal propeptides and progresses in a zipper-like fashion through the triple-helical domain, with the folding of the N-propeptide occurring last [51,52]. Transport to the Golgi follows and is coordinated by the formation of a COPII-dependent vesicle, which collects secretory cargo by the employment of the Sec23-Sec24 complex and other Sec proteins [53,54]. Once secreted into the extracellular matrix, the N- and C-propeptides are cleaved by peptidases and fibrillogenesis ensues for the fibrillar collagens [55].

Numerous proteins contribute to the biosynthesis of collagens in the rER. Chaperone proteins, such as GRP78 (BiP/HSPA5), GRP94, protein disulfide isomerase (PDI), calreticulin, calnexin, and cyclophilin B (CypB), nonspecifically bind elongating polypeptides at exposed hydrophobic residues, to prevent premature folding [49]. Ultimately, although chaperones may ensure that proper folding can take place, folding itself is directed by the thermodynamic interactions of the nascent polypeptide within the aqueous milieu of the ER lumen. In the presence of a nonsynonymous gene mutation, whereby an amino acid with a functionally different side chain is encoded, the biosynthesis and folding of collagens may be interrupted or prevented. Additionally, mutations in chaperone proteins or other resident rER proteins may also delay or disrupt the folding process. Such mutations can be pathogenic in two ways: (1) the absence of a particular collagen from the ECM may alter the structural integrity of a tissue; and (2) the aggregation of unfolded or misfolded proteins in the rER may induce UPR signaling, with apoptosis of the cell being a possible outcome.

## 6. The Unfolded Protein Response Has an Essential Role in Chondrogenesis

Perhaps paradoxically, studies that have examined the effects of knocking out key UPR genes have demonstrated that similar chondrodysplasic phenotypes result for those produced by UPR signaling in response to misfolded proteins. Although UPR signaling mediates the response to unfolded or misfolded proteins in the ER, it also has a physiological role in the maturation of chondrocytes during endochondral ossification. Each stage of chondrocyte maturation can be characterized by the expression of distinct ECM proteins and transcription factors. A necessary growth factor expressed by chondrocytes throughout chondrogenesis, bone morphogenetic protein 2 (BMP2), is an activator of UPR signaling in both chondrocytes and osteoblasts [56] (Figure 1). *BMP2* knockout mice display a severe chondrodysplasia phenotype and a disorganization of growth plate chondrocytes (Table 2) [57]. How BMP2-mediated induction of UPR signaling contributes to chondrocyte maturation and proliferation is still being elucidated. What is known about BMP-induced UPR signaling in chondrocytes is depicted in Figure 1. As chondrocytes are highly secretory and the maturation of the growth plate depends on the fidelity of the secretion of ECM proteins, physiological UPR signaling is likely to be important for the expedience of protein folding and secretion.

X-box binding protein 1 (XBP1) is a transcription factor located downstream of the IRE1 arm of the UPR, that is expressed by both proliferating and hypertrophic chondrocytes. In addition to the activation of XBP1 that occurs downstream of IRE1 activity, BMP2-induced Smad4 is a transcriptional activator of XBP1 [61]. XBP1 exists in both unspliced (XBP1-U) and spliced forms (XBP1-S), with the splicing of XBP1 mRNA performed by IRE1 under UPR activation [62]. XBP1S is expressed throughout the growth plate during chondrocyte differentiation, but the outcomes of chondrocyte-specific overexpression and ablation of XBP1S have differed among in vitro and in vivo reports. In the ATDC5 and BMSC cell lines, the adenovirus-mediated overexpression of XBP1S has been shown to increase the expression of the hypertrophy markers collagen X and Runx2 [61]. This may be suggestive of a role for XBP1S in initiating chondrocyte hypertrophy. However, recent in vivo analysis has not supported this conclusion. Cameron et al. (2015) constructed a mouse model in which XBP1 is functionally inactivated by the deletion of exon 2 (*Xbp1*^CartΔEx2^) [59]. Although mutant mice were born with shorter long bones and demonstrated delayed ossification when compared to WT mice, the phenotype of the mutants resolved by skeletal maturity. Although the work of Cameron and colleagues did not support a role for XBP1 in directing hypertrophy, it did suggest an alternative mechanism whereby XBP1 may appreciably impact chondrocyte proliferation. BrdU analysis of WT and *Xbp1*^CartΔEx2^ mice showed a modest hindrance in the proliferation of growth plate chondrocytes in the mutant. This could be due to altered Indian hedgehog (Ihh) and PTHrP signaling. As Ihh and PTHrP exist in a negative feedback loop to control the rate of hypertrophic differentiation [63], altering the expression of PTHrP may be responsible for delaying the relative pace of growth plate maturation in XBP1S knockouts, as observed both in vitro and in vivo.

BMP2 also induces the expression of the UPR transcription factor, activating transcription factor 4 (ATF4) [56]. ATF4 expression is under the control of PERK-eIF2a activation [33] and is expressed by all growth plate chondrocytes during development [58]. ATF4^−/−^ mice display a chondrodysplasia phenotype characterized by short stature, delayed endochondral ossification, a lengthened hypertrophic zone with fewer hypertrophic chondrocytes, and a disorganization in the columnar structure of the growth plate (Table 2). ATF4 is essential for the proper maturation of the growth plate. ATF4 has been shown to bind directly to the promoter of *IHH* and activate its transcription [58]. Recently, BMP2 has also been shown to induce COX-2 signaling in chondrocytes, leading to the PGE2-mediated phosphorylation of ATF4 [64]. As ATF4 has multiple phosphorylation sites, the significance of this event remains unclear.

The SRY-related transcription factor Sox9 is both necessary and sufficient for chondrogenesis and is also induced by BMP2 [65,66]. Indeed, the limb bud-specific inactivation of Sox9 prior to MSC condensation results in the absence of both cartilage and bone [67]. Sox9 induces the expression of Sox5 and Sox6, and activates the transcription of a number of collagen genes, including *COL2A1*, *COL11A2*, *COL9A1*, *COL9A2*, *COL27A1* [5], and possibly *COL5A1* and *COL11A1* [68]. Additionally, Sox9 binds to the promoter region of *BBF2H7*, a novel ATF6 homolog preferentially expressed by chondrocytes, and activates its transcription [69]. Like ATF6, BBF2H7 is proteolytically cleaved by the site-1 and site-2 proteases (SKI-1 and SKI-2) [60]. The C-terminal fragment of BBF2H7 is secreted into the ECM and promotes Hedgehog signaling via interaction with Ihh [70], while the N-terminus translocates to the nucleus to activate the transcription of Sec23a [60]. Sec23a is essential for the ER-to-Golgi export of proteins destined for secretion [53,71]. In BBF2H7^−/−^ mice, chondrocyte proliferation is severely perturbed (Table 2), and this is likely to be due to the insufficient secretion of ECM proteins required for chondrocyte maturation in the growth plate [60]. Interestingly, BBF2H7 also appears to have a key role in preventing UPR-induced apoptosis in chondrocytes [72]. This could explain earlier findings in metaphyseal chondrodysplasia, Schmid type (MCDS) chondrocytes, which do not undergo apoptosis despite elevated CHOP expression [48,73].

It remains unclear what distinguishes physiological and pathological UPR signaling in chondrocytes. UPR signaling is essential to chondrogenesis, as evidenced by the severe chondrodysplasias of UPR transducer-knockout mice (Table 2). However, in chondrodysplasias that involve UPR signaling in response to a mutant ECM gene, the relative contributions of pathological UPR signaling and the absence of a necessary ECM protein to disease phenotype are unknown (for review, see [39]). It appears that the timing of UPR coordination may be a differentiating variable. UPR signaling in chondrogenesis is a transient event, while UPR signaling in response to a consistently misfolded protein is sustained. Indeed, sustained activation of the UPR-induced transcription factor ATF4 may consequentially induce persistent Ihh signaling [58]. Although Ihh may induce PTHrP signaling to inhibit hypertrophy, it has also been shown to act independently of PTHrP to promote hypertrophy [74]. Furthermore, the role of Ihh signaling in the adult skeleton is unclear and conflicting hypotheses have been postulated [75]. Interestingly, recent evidence indicates that Ihh signaling in mature articular cartilage corresponds with the severity of OA [76,77], inhibiting Ihh signaling appears to improve the condition [75], and Ihh signaling in OA cartilage involves the upregulation of type X collagen and MMP-13 [77]. Whether increased Ihh signaling is seen in the articular cartilage of individuals with other chondrodystrophies such as Stickler syndrome has not been determined.

The pathophysiology of several chondrodysplasias resulting from mutations in collagens features robust UPR involvement. Skeletal defects and early-onset osteoarthritis (OA) present indiscriminately among many chondrodystrophic patients. Both endochondral bone development and joint health depend upon the structure and stability of cartilage. Cartilage is comprised of a fibrillar network of type II, XI, and IX collagens. The collaboration of these three collagens is essential for cartilage form and function. As mutations in the genes of any of these three collagens can cause Stickler syndrome, it is likely that the disruption of the cartilage fibrillar network underlies much of the Stickler phenotype.

## 7. Osteoblast Physiology and Altered Bone Mineralization in Chondrodystrophies and Early Onset OA

Many of the genes implicated in chondrodystrophies and early onset OA are expressed not only by chondrocytes, but also in cells of the developing inner ear (for review, see [78]), the vitreous [79], heart valves [80], and by osteoblasts [81]. How ER stress, UPR, and apoptosis may manifest in osteoblasts as a result of mutations in collagens has not been fully characterized. *COL2A1*, *COL11A1*, *COL11A2*, *COL9A1*, *COL9A2*, and *COL9A3* are all expressed, to some degree and at some time period, by osteoblasts [82,83]. Undermineralization of bone in chondrodystrophy resulting from a *COL2A1* mutation has been reported [84], and the *COL11A1*-haploinsufficient cho/+ mouse exhibits dysregulated mineralization in long bones [25,85]. The latter observation is congruent with a report that COL11A1 is a negative regulator of osteoblast maturation and the antisense morpholino oligonucleotide-mediated knockdown of COL11A1 in C2C12 myoblasts accelerates osteoblast differentiation, as measured by ALP [81]. COL11A2 expression in osteoblasts is directly activated by Osterix [86], but its expression pattern and function have not been characterized in mammalian osteoblasts. In the Atlantic salmon, *COL11A2* is expressed throughout the notochord in early stages of development, but quickly becomes confined to the segmented, non-mineralizing regions of the notochord, suggesting a similar role for COL11A2 in inhibiting mineralization [87]. COL9A1 may have a role in postnatal bone maintenance, as *COL9A1*^+/−^ and ^−/−^ mice develop osteoporosis in association with an increased osteoclast number and activity in trabecular bone [88]. Additionally, mutations in *COL9A1*, *COL9A2*, and *COL9A3* may cause multiple epiphyseal dysplasia, which is characterized by abnormal ossification of the epiphyses [89]. Presently, the specific roles of COL9A2 and COL9A3 in osteoblasts have not been elucidated.

Even at the clinical level, data on the bone health of patients with chondrodystrophies and early onset OA is scarce. The first report to describe the undermineralization of bone provided clinical evidence of reduced bone mass and bone turnover [84]. Subsequent radiographic evidence has demonstrated that patients may form large osteophytes (bone spurs) in narrowing osteoarthritic joints [90]. Osteoblasts within the osteophytes of patients with non-syndromic OA secrete the inflammatory cytokines IL-6 and IL-8, as well as MMP-13, and the expression of these factors increases upon mechanical stress [91]. MMP-13 is also expressed by articular chondrocytes in OA cartilage and contributes to the degradation of type II collagen. Understanding how osteophytes form and function may better inform our understanding of how precocious OA develops in individuals with chondrodysplasias.

When approaching questions concerning the state of osteoblasts in chondrodystrophies, it is important to note that osteoblasts, like chondrocytes, undergo BMP2-induced UPR activation during differentiation. Multiple events in osteoblastogenesis are attributed to BMP2 signaling, including the induction of Runx2, alkaline phosphatase (ALP), bone sialoprotein (BSP), osteocalcin, and COL1A1 [92,93]. The transcription factor Osterix (Osx/Sp7), a member of the Sp1 family of transcription factors, promotes mineralizing factors in osteoblasts and is transcriptionally activated by BMP2-induced XBP1S. In MC3T3-E1 osteoblasts, both BMP2 and thapsigargin (an ER stress inducer) promote Osx transcription in an XBP1S-dependent manner, illustrating that ER stress alone is sufficient for Osx induction [94]. If osteoblasts undergo additional ER stress in response to mutant collagen, increased Osx activity may result in inappropriate mineralization. Osx null mice display severe defects in bone mineralization and lack the expression of several markers for osteoblast differentiation, including osteocalcin, BSP, osteonectin, and osteopontin [92]. Although Osx is best known for its regulatory roles in osteoblast differentiation, the chondrocyte-specific ablation of Osx also impairs endochondral ossification and chondrocyte differentiation [95]. Also under the control of BMP2 signaling is OASIS, an ATF6 homolog that is highly expressed in osteoblasts and is structurally similar to BBF2H7. OASIS is a transcriptional activator of *COL1A1* [96]. Murakami and colleagues reported a 30%–40% decrease in *COL1A1* expression in the calvaria of OASIS^−/−^ mice. It is clear that BMP2-induced UPR signaling is necessary for the development of bone, so an understanding of how pathological UPR signaling may affect osteoblasts needs to be predicated on that necessity.

## 8. Stickler Syndrome

Stickler syndrome (ORPHA828), also known as hereditary progressive arthro-ophthalmopathy, is an inherited and progressive chondrodysplasia that is genetically and phenotypically heterogeneous. Individuals with Stickler syndrome may present clinically with short stature, craniofacial underdevelopment, sensorineural hearing loss, myopia, or precocious osteoarthritis [97]. While the UPR is involved in some collagen disorders, as discussed here, it is unclear to what extent these findings can be generalized. Mutational specificity and context must be taken into consideration when investigating the contribution of specific mutations within collagen molecules and their connection to ER stress and UPR, and how that may contribute to the development of chondrodysplasias and OA.

To date, multiple mutations in the collagen-encoding genes *COL2A1*, *COL11A1*, *COL11A2*, *COL9A1*, *COL9A2*, and *COL9A3* have been associated with six genetically distinct types of Stickler syndrome [15,98,99,100,101,102,103]. Types I-III, resulting from mutations in *COL2A1*, *COL11A1*, and *COL11A2*, are inherited in an autosomal dominant manner and account for the majority of occurrences of the disorder. Types IV-VI have been more recently characterized and display an autosomal recessive mode of inheritance. Although phenotypes vary widely among Stickler syndrome patients, only a few significant differences have been established between the six genetically-determined types. The sum prevalence of Stickler syndrome at birth is approximately 1/7500. It is estimated that 80%–90% of all Stickler syndrome cases are associated with pathological variants in *COL2A1* (Type I), 10%–20% of cases are *COL11A1*-related (Type II), and Types III-VI are rare and of an unknown prevalence [97].

Since Stickler syndrome was first described by Gunnar B. Stickler in 1965 [104], research efforts have largely been directed toward elucidating the genetic mutations that give rise to Stickler syndrome and the characterization of the phenotypes resulting from these mutations. While there are currently no disease-modifying therapies available for individuals with Stickler syndrome, there has been a shift of attention toward understanding the underlying molecular events implicated in the progression of the disease. Mutations in all of the Stickler-associated genes are also implicated in non-syndromic early-onset osteoarthritis (OA) [14], which may inform our understanding of how early-onset OA arises in Stickler syndrome. Additionally, Stickler syndrome shares many phenotypic characteristics with other chondrodysplasias, and some disease mechanisms may be shared among the congenital chondrodysplasias.

The underlying pathogenesis of Stickler syndrome is known to be presumed haploinsufficiency, due to the nonsense mediated degradation of mRNA from the mutant allele as a result of nonsense mutations or frameshifts, causing a premature termination codon. In these cases, misfolded proteins do not result because they are not translated, and thus, a UPR is not be induced. This is the case for the majority of Stickler mutations in *COL2A1*, which does not support an argument for the role of UPR as a significant pathological driver for the majority of Stickler cases associated with COL2A1.

While mutations in the predominant fibrillar collagen genes such as *COL2A1* that lead to haploinsufficiency due to the nonsense mediated degradation of mRNA from the mutant allele as a result of nonsense mutations or frameshifts, causing a premature termination codon may be unlikely to result in ER stress and UPR for major fibrillar collagens, this may not be the case for the minor fibrillar collagen α chains COL11A1 and COL11A2, for which the unique function and molecular mechanism they serve are less well understood. As regulators of fibril assembly, their function in the extracellular matrix may be preceded by functions during the assembly of collagens within the endoplasmic reticulum, playing the role of a positive regulator of folding and intermolecular interaction. Under these circumstances, it is feasible for mutations within *COL11A1* or *COL11A2* to result in ER stress and UPR. Recent evidence indicates that mutations in *COL11A1* result in the ER retention of secreted proteins, as demonstrated by ER dilation in mouse models carrying a mutation in the *Col11a1* locus, as well as the induction of ERAD, as evidenced by the upregulation of mRNAs encoding key regulators of the ERAD pathway upon knockdown of *COL11A1* expression in cell culture C28/I2 and ATDC5 cells from our laboratory.

## 9. Therapeutic Targets for the Modulation of UPR in Cartilage Pathophysiology

Future directions in chondrodysplasia therapeutic approaches may focus on mediators of ER stress and the UPR. Development of pharmacological interventions for chondrodysplasias caused by ER stress would have a significant impact on the frequency with which surgical procedures are needed. Therapies may include (1) inhibition of ER stress; (2) activation of protein folding pathways by chaperone proteins; (3) enhancement of ER-associated degradation; (4) inhibition of inflammatory modulators; (5) treatment with chemical chaperones; and (6) RNAi treatment to reduce the level of expression of the mutated collagen in the case of collagenopathies. While a link between osteoarthritis and the unfolded protein response has been described by several laboratories, osteoarthritis is not likely to be the result of an overall activation of ER stress [105]. A decrease in the PERK pathway has been observed in osteoarthritic chondrocytes with an associated decrease in *COL2A1* expression, contributing to the degradation of the extracellular matrix. PERK may therefore be considered as a therapeutic target for the treatment of osteoarthritis [105]. Broadening the scope beyond osteoarthritis, ER stress and the UPR may also play a role in the development and progression of rheumatoid arthritis [106].

## 10. Discussion

Early onset osteoarthritis is a frequent hallmark of chondrodystrophies resulting from disturbance in homeostasis. Understanding the mechanisms that underlie chondrocyte apoptosis during endochondral ossification in the growth plate has a potential therapeutic application for early onset osteoarthritis. ER stress, ERAD, and UPR play an essential role in homeostasis during the growth and maturation of the skeleton; however, conditions in which the protein folding load outweighs the folding capacity of the ER lead to cellular dysfunction and death. Of particular relevance to endochondral bone formation are the collagens. The collagens are structural, extracellular matrix (ECM) proteins that fulfill differing roles throughout the course of chondrogenesis. Deleterious polymorphisms in collagen-encoding genes prevent the proper folding, assembly, or function of collagens. Collagen misfolding has consequences for not only the extracellular microenvironment, but also for the intracellular homeostasis of chondrocytes and osteoblasts. The UPR functions to restore homeostasis by upregulating chaperone proteins to assist with folding, preventing the translation of non-UPR proteins, and initiating apoptosis when resolution is unlikely. UPR signaling has dually essential and detrimental roles in the bone development of individuals with congenital collagenopathies and chondrodysplasias.

Precocious OA associated with chondrodystrophies may follow similar signaling cascades in its progression as age-related OA. OA manifests in the majority of patients with chondrodysplasias prior to 30 years of age [16]. OA may result from an enlarged collagen fibril diameter, increasing the potential for articular chondrocyte cell-surface Ddr2 to bind type II collagen and activate MMP-13 transcription. However, many pathways of OA progression also involve MMP-13 signaling [24]. A better understanding of OA progression in chondrodystrophies will inform our understanding of how late-onset OA progresses.

*The role of AGE/RAGE signaling in early-onset OA*. When subjected to OA-inducing surgery, four week old RAGE-knockout mice experienced a milder OA compared to WT mice [23]. AGE formation in articular cartilage is positively correlated with age, but the upstream contributors to AGE formation in OA remain unknown. The altered microarchitecture of articular cartilage in chondrodystrophies may contribute to the formation of AGEs.

*Consequences of pathological UPR signaling in chondrocytes.* Chondrocytes express the ATF6 homolog, BBF2H7, which may prevent CHOP-mediated apoptotic signaling [72]. In models of MCDS, UPR activation in hypertrophic chondrocytes results in the dedifferentiation of chondrocytes to a prehypertrophic state [48,107]. Whether pathological UPR signaling results in altered differentiation has not been explored in other chondrodysplasias or other stages of chondrocyte maturation.

Numerous mutations in *COL2A1* have been shown to induce UPR signaling in chondrocytes [45]. However, it is unclear whether these mutations are representative of those experienced by individuals with mutations in other collagen genes and other ECM proteins of cartilage [108,109,110]. Furthermore, it has been suggested that the intracellular retention of either COMP or one of its binding partners may result in the co-retention of the other [111]. The retention of COMP in the rER lumen results in a novel form of oxidative stress in the chondrodysplasias, multiple epiphyseal dysplasia (MED) and pseudoachondroplasia (PSACH) [112,113].

## 11. Conclusions

Early onset osteoarthritis is common in chondrodystrophies, resulting from disturbances in homeostasis. ER stress induces one or more branches of the UPR signaling pathways to reestablish homeostasis. If homeostasis is not reestablished, one potential outcome is apoptosis. ER stress, UPR, and ERAD may represent therapeutic targets for the modulation of skeletal disorders that lead to osteoarthritis.

## Figures and Tables

**Table 1:** Mutations in collagen genes that may induce ER stress/UPR and lead to dysplasia.

Collagen Chain	Mutation Described	Dysplasia Resulting from Mutation(s)	Observed	References
*COL1A1*	Aga2/+ mouse; C-propeptide mutation in col1a1	Osteogenesis imperfecta	UPR leads to osteoblast apoptosis	[44]
*COL2A1*	*Col2a1* p.Gly1170Ser mouse	Chondrodysplasia	UPR leads to apoptosis of chondrocytes prior to hypertrophy; no hypertrophic zone formed	[45]
	Dmm/+ mouse; C-propeptide mutation in col2a1	Chondrodysplasia with early-onset OA	UPR leads to articular chondrocyte apoptosis, contributes to early OA	[28]
*COL3A1*	15 human patients with unique col3a1 mutations	Ehlers-Danlos type IV	Retention of Col3a1 procollagen in ER; distension of rough ER	[46]
*COL5A1*	21 patients harboring unique mutations in Col5a1 or Col5a2	Classic Ehlers-Danlos	Variability in collagen fibril diameter, collagen cauliflowers (aggregates), and dilated ER	[47]
*COL5A2*				
*COL10A1*	Transgenic mouse: 13 bp deletion within NC1 domain (13del)	Metaphyseal chondrodysplasia, type Schmid (MCDS)	UPR induction in hypertrophic chondrocytes and dedifferentiation to a pre-hypertrophic state	[48]

**Table 2:** Knockout of UPR-related genes that may result in a chondrodysplasia phenotype in mice.

Gene Symbol	Gene Name	Function in Chondrocytes/Osteoblasts	Knockout Phenotype	Reference
*ATF4*	Activating transcription factor 4	Induces CHOP activity (pro-apoptotis)	Severe skeletal defects, delayed ossif., short stature/limbs, disorganization of growth plate chondrocyte columns	[58]
*BMP2*	Bone morphogenetic protein 2	Induces physiological UPR in chondrocytes and osteoblasts	Severe chondrodysplasia, disorganization of growth plate chondrocytes	[57]
*XBP1*	X-box binding protein 1	May promote chondrocyte proliferation	Delayed ossification that resolves by maturity	[59]
*BBF2H7*	BBF2 human homolog on chromosome 7	Sequence similarity to ATF6 (in CREB/ATF family); expressed in chondrocytes; targets Sec23a in ER-to-Golgi transport	Severe chondrodysplasia, proliferating chondrocytes show abnormal ER distension	[60]

## Abbreviations

AGEs	Advanced glycation end-products
ALP	Alkaline phosphatase
ATF4	Activating transcription factor 4
ATF6	Activating transcription factor 6
BBF2H7	BBF2 human homolog on chromosome 7
BMP2	Bone morphogenetic protein 2
BSP	Bone sialoprotein
*cho*	Chondrodysplasia
CHOP	C/EBP homologous protein
COMP	Cartilage oligomeric matrix protein
CypB	Cyclophilin B
DMM	Disproportionate micromelia mouse
DDR2	Discoidin domain receptor-2
ECM	Extracellular matrix
ER	Endoplasmic reticulum
FACIT	Fibril-associated collagen with interrupted triple helices
GRP78	Glucose-regulated protein, 78 kDa (also known as BiP/HSPA5)
HtrA1	High temperature requirement protease A-1
IHH	Indian hedgehog
IRE1	Inositol-requiring endonuclease 1
LH	Lysyl hydroxylases
MCDS	Metaphyseal chondrodysplasia, type Schmid
MED	Multiple epiphyseal dysplasia
MMP-13	Matrix metalloproteinase-13
OA	Osteoarthritis
OSX	Osterix (Osx/Sp7)
P3Hs	Prolyl 3-hydroxylases
P4Hs	Prolyl 4-hydroxylases
PERK	PKR-like endoplasmic reticulum kinase
PDI	Protein disulfide isomerase
PSACH	Pseudoachondroplasia
PTHrP	Parathyroid hormone related peptide
RAGE	Receptor for AGEs
RER	Rough endoplasmic reticulum
UPR	Unfolded protein response
VR	Variable region
XBP1	X-box binding protein 1
XBP1S	Spliced X-box binding protein 1
XBP1U	Unspliced X-box binding protein 1
